# Stages of Behavioral Change for Reducing Sodium Intake in Korean Consumers: Comparison of Characteristics Based on Social Cognitive Theory

**DOI:** 10.3390/nu9080808

**Published:** 2017-07-27

**Authors:** So-hyun Ahn, Jong Sook Kwon, Kyungmin Kim, Hye-Kyeong Kim

**Affiliations:** 1 Department of Food Science and Nutrition, The Catholic University of Korea, Bucheon 14662, Korea; sohyunahn1123@nate.com; 2 Department of Food and Nutrition, Shingu College, Songnam 13174, Korea; jskwon@shingu.ac.kr; 3 Department of Food and Nutrition, Baewha Women’s University, Seoul 03039, Korea; kyungmkim@baewha.ac.kr

**Keywords:** stage of behavioral change, reducing sodium intake, consumer, social cognitive theory

## Abstract

High sodium intake increases the risk of cardiovascular disease. Given the importance of behavioral changes to reducing sodium intake, this study aims to investigate the stages of change and the differences in cognitive and behavioral characteristics by stage in Korean consumers. Adult participants (*N* = 3892) completed a questionnaire on the stages of behavioral change, recognition of social efforts, outcome expectancy, barriers to practice, nutrition knowledge and dietary behaviors, and self-efficiency related to reduced sodium intake. The numbers of participants in each stage of behavioral change for reducing sodium intake was 29.5% in the maintenance stage, 19.5% in the action stage, and 51.0% in the preaction stage that included the precontemplation, contemplation, and preparation stages. Multiple logistic regression showed that the factors differentiating the three stages were recognizing a supportive social environment, perceived barriers to the practice of reducing sodium intake, and self-efficacy to be conscious of sodium content and to request less salt when eating out. Purchasing experience of sodium-reduced products for salty foods, knowledge of the recommended intake of salt and the difference between sodium and salt, and improving dietary habits of eating salted fish, processed food, and salty snacks were factors for being in the action stage versus the preaction stage. These findings suggest that tailored intervention according to the characteristics of each stage is helpful in reducing sodium intake.

## 1. Introduction

High dietary sodium intake is a major risk factor for hypertension that can induce cardiovascular disease and stroke [1,2] and is associated with an increased risk of renal disease, osteoporosis, and gastric cancer [3]. It has been reported that effective sodium-reduction programs not only improve public health, but are also cost effective [4,5]. In Korea, the food industry and government have made considerable efforts to develop sodium-reduced products and to raise public awareness through campaigns, public advertisements, and the labeling of sodium content in foods. Consequently, the average sodium intake in Korea has shown a decreasing trend since 2005. The average daily sodium intake was 3890 mg in 2014, which was reduced by 25% from 5256 mg in 2005, but it is still almost double the recommended daily intake [6].

Although public awareness is an important component of a successful salt-reduction initiative [7,8], awareness of the importance of sodium reduction is not always associated with behavioral change. The majority of dietary sodium in Korea is added to food during cooking and eating at the table [9], which suggests the importance of consumer education to change behavior to reach the recommended sodium-intake level. A transtheoretical model of behavioral change is useful to assess whether consumers are oriented toward change. According to the model, there are five stages of behavioral change that people move through in adopting a health-related behavior: precontemplation, contemplation, preparation, action, and maintenance [10].

Precontemplation is the stage in which people do not intend to change their behavior in the next six months and are not aware that their behavior is problematic [10]. People in the contemplation stage express an intention to make a change at some time but not soon; they are aware of the pros of changing and acutely aware of the cons. Preparation is the stage in which people intend to take action within the next month or may already have begun some significant steps toward behavioral change. The action stage is defined by consistent practice in the recent past, while the maintenance stage is typified as consistent practice for more than six months. Although this model has been applied to the individual for a variety of health behaviors [10,11], it can be used to assess the health behavior status of community members and to measure the effects of interventions [12]. A recent online survey conducted in eight countries reported that about one third of the population was not interested in reducing their salt intake (precontemplation stage), and only 39% of the population was in the action and maintenance stages [13]. However, there has been no report on the status of behavioral change with respect to reducing sodium intake in Korea. 

Social cognitive theory is a comprehensive framework for understanding health-related behaviors and changing behaviors [14]. The theory proposes that behavior is a function of the aspects of the environment and of the person, all of which are in constant interaction. Personal factors for understanding behavior include skills and knowledge to perform the behavior, self-efficacy, and the outcome expectancy of the behavior. Environmental aspects influence the individual’s behavior by providing appropriate modeling for learning the behavior and available materials to use [15]. It is necessary to understand the relationship between the stage of change and personal cognitive and behavioral characteristics to develop effective intervention for consumers. However, no previous studies have addressed the differences in reducing sodium intake by stages of behavioral change. 

The objective of this study was to examine the status of behavioral change in reducing sodium intake and to describe the association between the stages of change and cognitive and behavioral factors in Korean consumers.

## 2. Subjects and Methods

### 2.1. Study Design and Participants

A nationwide cross-sectional survey was performed through a local network of the Korean National Council of Consumer Organizations. The regional distribution of the Korean population in 2011 was considered in the sampling. Participants aged at least 18 years were recruited through announcements to consumers using phone calls, e-mails, local newspaper advertisements, school newsletters, and Internet boards. All participants gave their informed consent for inclusion before they participated in the study. This study was conducted in accordance with the Declaration of Helsinki, and the protocol was approved by the Ethics Committee of the Catholic University of Korea (1040395-201705-02). The survey was conducted from 30 June 2011 to 31 October 2011.

### 2.2. Questionnaire

A self-administered questionnaire was developed based on previous studies [16,17,18], and face validity was established by experts. The questionnaire consisted of the following three sections: demographic information, questions for classifying the stage of behavioral change, and cognitive and behavioral factors related to reducing sodium intake (see in Supplementary Material). 

#### 2.2.1. Stage of Behavioral Change

The stage of behavioral change in reducing sodium intake was assessed by an algorithm with separate questions. Participants were asked, ‘Are you currently practicing a low sodium diet? (a) yes, more than six months; (b) yes, but less than six months; (c) no’. Participants who responded ‘(a) yes, more than six months’ were classified in the maintenance stage; those who responded ‘(b) yes, but less than six months’ were classified in the action stage; and those who responded ‘(c) no’ were asked an additional question to differentiate their stages. The question was ‘Do you intend to make changes to reduce sodium intake in the near future or in the following month? (a) yes; (b) no, but in the next six months; (c) no, I haven’t thought about it’. If they responded ‘(a) yes’, they were classified in the preparation stage; if they responded ‘(b) no, but in the next six months’, they were classified in the contemplation stag; and those who responded ‘(c) no, I haven’t thought about it’ were classified in the precontemplation stage.

#### 2.2.2. Cognitive and Behavioral Factors

Consumer cognition related to reducing sodium intake was assessed according to four categories: recognition of supportive environment and experience in purchasing sodium-reduced foods, positive outcome expectancy and barriers to reducing sodium intake, perception and self-efficacy of reducing sodium intake, and nutrition knowledge. The list of positive outcome expectancy to low sodium intake was suggested, and participants were asked to select three main outcomes. The barriers to reducing sodium intake were suggested as follows: ‘bad taste’, ‘hard to prepare and cook’, ‘limitation in choosing the food, menu, and restaurant’, ‘limited information, knowledge, and skills to practice’, ‘limitation to social relationship when dining with family or friends’, ‘preference for broth dishes (soup, stew)’, and ‘preference for kimchi, salted fish, and fermented sauces’. Perception and self-efficacy of reducing sodium intake were evaluated using eight questions, including consciousness of saltiness in food, willingness to select fresh food rather than processed or seasoned food, requesting less salt when eating out, and interest in low-sodium recipes. Nutrition knowledge was evaluated using 10 questions, including a conceptual understanding of sodium and salt, the health risks of excess sodium intake, the recommended daily intake of sodium, the physiological functions of sodium, the benefits of reducing sodium intake, high sodium foods, and nutrition labeling. In addition, dietary behavior related to sodium intake was assessed using 13 questions, including checking the sodium content in nutrition labeling, the frequency of eating out and eating high sodium foods, and habits of eating broth and adding salt to dishes. Participants were asked to respond ‘yes’, ‘no’, or ‘I don’t know’ to the questions on nutritional knowledge. Barriers, perception and self-efficacy, and dietary behavior were rated with a five-point Likert scale, ranging from 1 (strongly disagree) to 5 (strongly agree). In the present study, Cronbach’s alpha for barriers, perception and self-efficacy, nutrition knowledge, and dietary behavior ranged from 0.72 to 0.78, indicating good internal consistency in the responses.

### 2.3. Statistical Analysis

Data were analyzed using the SAS (version 9.3, SAS Institute, Inc., Cary, NC, USA) package program. Age, body mass index (BMI), and Likert scores were reported as means ± standard deviations. Means of the Likert scores were compared by analysis of covariance (ANCOVA) according to the stage of behavioral change. All categorized data were analyzed using chi-square tests. Multiple logistic regression was performed to examine the relationship between the stage of change and cognitive and behavioral factors in reducing sodium intake. A *p*-value of < 0.05 was considered statistically significant.

## 3. Results

### 3.1. Participant Demographics

A total of 3892 participants were included in the study after people who did not answer to the question to assess their stage of behavioral change were excluded (inclusion rate 89%). The general characteristics of the participants are shown in Table 1. The participants were aged 18 to 85 years, with a higher proportion in their 40s and 50s. Most of the participants were women (94.8%). Approximately 45% of the participants were college graduates or had higher levels of education.

When the participants were classified according to the stage of behavioral change, 29.5% were in the maintenance stage and 19.5% were in the action stage. The proportions of participants in the precontemplation stage, contemplation stage, and preparation stage were 23.3%, 24.0%, and 3.7%, respectively. These three groups were combined for analysis because there were relatively small numbers of participants in the preparation stage, and understanding the characteristics of those in the preaction stages is necessary to move forward to the action stage. Thus, five stages of behavioral change were reduced to three groups: maintenance (M), action (A), and preaction (P). Significant associations were found between demographic variables and an individual’s stage of change. Thus, analyses of the association between cognitive and behavioral factors and the stages of change were adjusted by demographic variables such as age, gender, BMI, education level, and monthly income level.

### 3.2. Recognition of Supportive Environment and Experience of Purchasing Reduced Sodium Foods

The percentage of participants who recognized social efforts for reducing sodium intake through campaigns and nutritional education was 82.5% in the maintenance stage group; furthermore, the percentages were 74.9% and 54.7% in the action stage and preaction stage groups, respectively. Similarly, the largest proportions of participants recognizing sodium content labeling on processed food were in the order of the maintenance (74.4%), action (66.4%), and preaction (58.8%) stage groups. Recognizing social efforts and sodium content labeling on processed foods increased the odds of being in the action stage rather than the preaction stage by about 2.3 fold and 1.8 fold, respectively. Further, these two variables increased the odds of being in the maintenance stage versus the action stage significantly (Table 2).

The most frequently purchased reduced-sodium products were ham (36.7%), salt (32.8%), and cheese (26.7%). The experience rates of purchasing sodium-reduced foods were different among the three stage groups. Participants who had purchased low-sodium salt, sodium-reduced salted fish, low-sodium soy sauce, low-sodium ham, and low-sodium cheese had significantly higher odds of being in the action stage rather than the preaction stage. In addition, the purchasing experience of buying low-sodium cereal, low-sodium cheese, and low-sodium ramen increased the odds of being in the maintenance stage rather than the action stage (Table 2).

### 3.3. Positive Outcome Expectancy and Barriers to Reduce Sodium Intake

Table 3 presents the positive outcome expectancy and the barriers to reducing sodium intake that were statistically different among participants in the three stages of behavioral change. Participants perceived a decrease in blood pressure and an increase in the prevention of strokes and heart disease as the main benefits of reducing their sodium intake. Participants expecting a decrease in blood pressure as a reward for reducing their sodium intake had higher odds of being in the action stage versus the preaction stage. An expectation of cancer prevention was relatively low, but those who perceived the benefit had higher odds of being in the maintenance stage.

The proportions of participants who agreed to each barrier were the highest in the preaction stage followed, in order, by the action and maintenance stages. Having the following barriers reduced the odds of being in the action stage versus the preaction stage and the odds of being in the maintenance versus the action stage significantly: ‘limited information, knowledge, and skills’, ‘bad taste’, ‘limitation to social relationship when dining with family or friends’, and ‘time-consuming and inconvenient process of cooking and preparing’. This result indicates that overcoming these barriers is important for individuals to take action. On the other hand, both ‘preference for broth dishes’ and ‘preference for kimchi, salted fish, and fermented sauces’ reduced the odds of being in the maintenance stage only by about half (0.46 and 0.45, respectively), which suggests that overcoming these two barriers are critical factors to reach sustained behavioral change for reducing sodium intake.

### 3.4. Perception and Self-Efficacy on Reducing Sodium Intake

All the questions on perception and self-efficacy related to reducing sodium intake showed significant differences between the three stages of change (Table 4). The scores were highest in the maintenance stage, followed by the action stage and the preaction stage. ‘Willingness to buy fresh food rather than processed or instant food’ got the highest score in all three groups, while ‘unsatisfied feeling when eating foods with less salt’ had the lowest score. This indicates that an ‘unsatisfied feeling when eating foods with less salt’ was the hardest barrier to overcome for respondents.

‘Recognizing sodium content’, ‘not feeling unsatisfied when eating foods with less salt’, and ‘requesting less salt when eating out’ enhanced the odds of being in the action stage versus the preaction stage and the odds of being in the maintenance stage versus the action stage significantly. ‘Recognizing the sodium content’ enhanced the odds ratio by more than two fold for the action stage versus the preaction stage (odds ratio (OR); 2.2, 95% confidence interval (CI); 1.79–2.71) and for the maintenance stage versus the action stage (OR; 2.3, 95% CI; 1.77–2.96). On the other hand, the perception that ‘practicing a low-sodium diet will improve my health status’ was the only factor related to being in the maintenance stage rather than the action stage without differentiating between the preaction stage and the action stage. Participants with this perception had an odds ratio of 2.24 (95% CI; 1.32–3.81) for being in the maintenance stage versus the action stage.

### 3.5. Nutrition Knowledge Related to Sodium Intake

The rates of correct answers to most questions and the average scores of nutrition knowledge were the highest in the maintenance stage, followed by the action and preaction stages (Table 5). About 83% of participants knew that a sufficient intake of vegetables and fruits helps with sodium excretion. On the contrary, more than two thirds of participants did not know the difference between sodium and salt. Half the participants also did not know the recommended daily intake of sodium. The odds of being in the action stage were significantly enhanced when the participants knew the recommended daily intake of sodium and the difference between sodium and salt. This indicates that these concepts are important to taking action, although they are difficult. Notably, average scores of nutritional knowledge over six points enhanced the odds ratio of the maintenance stage versus the action stage without differentiating between the action stage and the preaction stage. Likewise, knowledge of the benefits of the sufficient intake of vegetables and fruits with regard to sodium excretion, using cooking methods to reduce sodium, the health risks of high sodium intake, the high sodium content in broth, and the physiological function of sodium was related to the enhanced odds of being in the maintenance stage.

### 3.6. Dietary Behavior Related to Sodium Intake

Table 6 presents the results of dietary behavior related to sodium intake that were statistically different among the three stages of behavioral change. Participants in higher stages of change showed more desirable dietary behavior in 11 out of 13 questions. Having good behavior in terms of checking the sodium content on nutritional labeling, not adding salt or sauce, the frequency of eating soup or stew, and a preference for grilled food over braised food with soy sauce enhanced the odds of being in the action stage versus the preaction stage and the odds of being in the maintenance stage versus the action stage significantly. On the other hand, improving dietary habits by not eating dried or salted fish, processed or instant food, salty snacks such as potato chips and crackers, and not frequently eating out was an important factor in being in the action stage rather than the preaction stage, without differentiating between the action stage and the maintenance stage. This was particularly true for participants avoiding processed or instant foods, who had an odds ratio of 1.93 (95% CI; 1.07–1.82) for being in the action stage versus the preaction stage. Eating plenty of fruits and vegetables was the only factor related to being in the maintenance stage rather than the action stage (OR; 1.99, 95% CI; 1.34–2.95) without differentiating between the preaction stage and the action stage.

## 4. Discussion

This study provides the status of behavioral change in reducing sodium intake among Korean consumers. A recently performed an online survey in eight countries and showed that the percentages of people in each of the behavioral stages of salt reduction were 58% of the people in the preaction stage, 13% in the action stage, and 28% in the maintenance stage, although there was a significant difference across the countries in the distribution of the stages of change [13]. Koreans had higher proportions of people in the action and maintenance stages, potentially due to the recent nationwide sodium-reduction initiative. In addition, Koreans are open to sodium reduction compared with other people in the international study, considering that 23.3% of the participants of this study and 34% of the international study participants reported no intention to make changes in sodium intake (precontemplation stage) [13]. This indicates that it is time to change the strategy for moving from awareness to action for sodium reduction, although there is still a need to raise awareness and interest for those in the precontemplation stage. 

This study examined cognitive and behavioral factors based on social cognitive theory according to the stages of behavioral change to assess the factors that were associated with taking action and maintaining the changes for reducing sodium intake. We classified responders into three categories for statistical analyses: the preaction, action, and maintenance stages. Multiple logistic regression analysis displayed meaningful factors differentiating the three stages. The comparison of participants in the action stage with those in the preaction stages was considered as determining the odds of ‘taking action to reduce sodium intake’, while the comparison of participants in the maintenance stage with those in the action stage reveals the odds of ‘maintaining changes for reducing sodium intake’.

Many studies highlight that attitudes, knowledge of sodium intake, and health beliefs are important for changing sodium intake [19,20]. In addition to these factors, the perception of the social environment related to sodium intake seems to be important to changing behavior because the proportion of meals eaten outside of the home is continuously increasing as is the nationwide promotion of reducing sodium. The World Health Organization (WHO) recommends the development of supportive environments that promote healthy food choices to reduce the sodium consumption of the general population [21]. Recognizing social efforts and sodium labeling on processed foods were important factors for being in the action and maintenance stages. Further, participants who had purchased sodium-reduced foods, especially those recognized as salty foods such as ham, salt, soy sauce, and salted fish were more likely to be in the action stage. Previous studies have reported that most participants were capable of estimating the salt content of salty foods, but they were unaware of the salt content of the usual processed foods [20,22,23]. Thus, it is reasonable that consumers bought sodium-reduced products for salty foods first in the action stage and then began to consider less salty processed foods such as cereals and ramen in the maintenance stage. A supportive environment influences people to change by providing models for change and available foods for reduced sodium consumption [15]. 

Outcome expectancy is known as the primary motivational variable to elicit a change in behavior. It was reported that people who were aware that sodium intake was associated with increased blood pressure were more likely to practice sodium reduction than those who were not aware (OR = 2.17, 95% CI; 2.01–2.34) [24]. Our result is consistent with the previous study, although the odds ratio of being in the action stage is weaker. The result that the expectation of cancer prevention enhanced the likelihood of being in the maintenance stage indicates the need to educate the populace on the diverse health-related benefits of reducing their sodium intakes. Indeed, better knowledge about the relationship between sodium intake and osteoporosis was related to being in the maintenance stage, as shown in the results of the nutritional knowledge test. This study revealed that overcoming barriers to practicing was associated with the differentiation of the three stages, but ‘preference for broth dishes’ and ‘preference for kimchi, salted fish, and fermented sauces’ were hard to overcome in the early action stage. The majority of Koreans’ sodium intake comes from fermented and salted traditional foods and soup-based meals [25]. These deeply rooted dietary habits are difficult to change but are critical to reaching the maintenance stage. Thus, more strategic intervention is needed such as developing various salt substitutes [26], fermentation technology to reduce sodium use, and reducing the size of the soup bowl.

Contrary to our expectations, the total nutritional knowledge score was not a differentiating factor between the preaction and action stages. Only knowledge on the recommended daily intake of salt and the difference between sodium and salt has a positive effect on the odds ratio of being in the action stage versus the preaction stage. The correct answer rate of these items was low, which is consistent with previous studies [13,19,27,28]. Lack of knowledge in these areas means that consumers are unlikely to be able to estimate their daily sodium intake and compare their intake with the recommended level. Indeed, it was reported that participants believed that their sodium intakes were equal to or less than the recommended level [24,29], despite strong evidence that the sodium intake of most populations exceeds the recommended level [30,31]. Therefore, it is evident that the recommended daily intake of sodium and the difference between sodium and salt should be focused on in the education of people in all the stages. The nutritional knowledge of the Korean consumer seems to be relatively high, considering a recent study that summarized the previous reports on the nutritional knowledge of the general population [32]. Detailed skills in practice are more effective for taking action in a situation in which the nutritional knowledge of the general population has reached a certain level. Actually, in our study, detailed dietary behaviors regarding low-sodium food selection and food preparation were different among the stages of change, and almost all of the desirable behaviors were associated with being in the action stage versus the preaction stage. It is known that behavioral change is a dynamic process that occurs in a sequential and cyclical order [33], which suggests the need for continuous education even for people in the action and maintenance stages.

In addition, self-efficacy is the primary resource for performing the behavior, considering that self-efficacy was more strongly related to intention to perform healthy eating practicing than was outcome expectancy [34]. Self-efficacy requires the ability to perform the behavior under a variety of circumstances, which suggests the need for nutrition education to improve skills and dietary behavior. Given the differences in cognition and behavior according to the stages, a tailored strategy, which is focused on motivating changes and raising self-efficacy, would be a promising approach. The results of this study suggest that educating individuals in detailed dietary behavior such as how to select low-sodium food would be more effective for those in the preaction stage, while it would be effective for those in the action stage to understand advanced nutritional knowledge such as the diverse health-related benefits of sodium reduction, tips to reduce sodium intake when cooking and eating, and the importance of a sufficient intake of fruits and vegetables.

This study has some limitations. We depended upon a self-administered questionnaire to obtain the results on the sample’s dietary behavior related to sodium intake. Self-reports are likely to be biased to social expectation and difficult to verify. However, the results correspond to the differences in perception of barriers to reducing sodium intake according to the stages. We modified the five-stage model to three stages to simplify the analysis. This division may have masked factors associated with a readiness to make changes in sodium reduction, although our main interest was in taking action and maintenance. In addition, compared with the general Korean population, the participants were predominantly women and over-representative of the over 40 age group, who tend to be more health conscious. Nonetheless, this study provides a valid estimate because the recruitment of participants was nationwide with geographic distribution and the residential local size of population was taken into consideration. 

In summary, the percentages of Korean consumers in each stage of behavioral change in order to reduce their sodium intake was 51.0% in the preaction stage, 19.5% in the action stage, and 29.5% in the maintenance stage. The factors associated with taking and maintaining action to reduce sodium intake were recognizing a supportive social environment, reducing barriers to practice, and enhancing self-efficacy. Therefore, campaigns that inform consumers of the health risks of high sodium intake and the establishment of a supportive environment, including sodium labeling, are effective for all consumers. In addition, there is a need for tailored education in purchasing, cooking, and eating according to the stages of behavioral change to reduce barriers and enhance self-efficacy.

## 5. Conclusions

The differences in cognitive and behavioral factors among the stages of behavioral change for reducing sodium intake in Korean consumers suggest the need of stage-matched intervention to reduce barriers and enhance self-efficacy for practicing low sodium diet, in addition to continual development of supportive social environment to raise public awareness.

## Figures and Tables

**Table 1:** Participants’ demographics.

	M (*N* = 1151)	A (*N* = 758)	P (*N* = 1983)	Total (*N* = 3892)	*p*-Value
Stage of change (%)	29.6	19.5	50.9	100.0	
Age	52.7 ± 10.4 ^(1)^^,a^	52.2 ± 11.1 ^a^	46.3 ± 11.8 ^b^	49.3 ± 11.7	<0.0001
BMI (kg/m^2^)	22.4 ± 2.5 ^b^	22.6 ± 2.6 ^a^	22.2 ± 2.6 ^b^	22.3 ± 2.6	0.0032
Age					
18–29	19 (1.7%) ^(2)^	18 (2.5%)	143 (8.1%)	190 (5.1%)	<0.0001
30–39	107 (9.8%)	82 (11.3%)	400 (20.8%)	589 (15.7%)	
40–49	259 (23.6%)	170 (23.5%)	614 (32.0%)	1043 (27.9%)	
50–59	427 (38.9%)	268 (37.0%)	497 (25.9%)	1192 (31.9%)	
60–69	229 (20.9%)	152 (21.0%)	206 (10.7%)	587 (15.7%)	
Over 70	56 (5.1%)	35 (4.8%)	49 (2.6%)	140 (3.8%)	
Gender					
Male	28 (2.5%)	26 (3.5%)	146 (7.5%)	200 (5.3%)	<0.0001
Female	1096 (97.5%)	719 (96.5%)	1797 (92.5%)	3612 (94.8%)	
Education level					
Middle school or less	148 (13.2%)	110 (15.0%)	186 (9.6%)	443 (11.7%)	<0.0001
High school	498 (44.4%)	395 (53.6%)	739 (38.2%)	1632 (43.0%)	
College or above	476 (42.5%)	232 (31.5%)	1009 (52.2%)	1717 (45.3%)	
Average monthly income					
Under $1000	116 (10.8%)	76 (11.3%)	141 (7.7%)	333 (9.3%)	0.0001
$1000~$2000	172 (16.0%)	127 (18.8%)	341 (18.6%)	640 (17.8%)	
$2000~$3000	252 (23.4%)	188 (27.9%)	456 (24.8%)	896 (25.0%)	
$3000~$4000	215 (20.0%)	141 (20.9%)	382 (20.8%)	738 (20.6%)	
$4000~$5000	171 (15.9%)	85 (12.6%)	316 (17.2%)	572 (15.9%)	
Over $5000	150 (13.9%)	58 (8.6%)	201 (10.9%)	409 (11.4%)	

M = Maintenance stage, A = Action stage, P = Preaction stage including the preparation, contemplation, and precontemplation stages, BMI = Body mass index. ^(1)^ Mean ± SD: Mean values with different superscripts are significantly different between the groups at α = 0.05 as determined by Duncan’s multiple range test after one-way ANOVA; ^(2)^ *N* (%): by chi-square test among the groups according to the stage of change.

**Table 2:** Recognition of supportive environment and purchasing experience of reduced sodium foods.

	M	A	P	Total	*p*-Value ^(1)^	A vs. P		M vs. A	
						OR ^(2)^	95% CI	OR	95% CI
Recognition of social efforts for reducing sodium intake									
Yes	941 (82.5%) ^(3)^	569 (74.9%)	1080 (54.7%)	2590 (66.8%)	<0.0001	2.25 *	1.83–2.77	1.58 *	1.24–2.02
Recognition of sodium labeling on processed foods									
Yes	741 (74.4%)	485 (66.4%)	1111 (58.8%)	2418 (64.9%)	<0.0001	1.83 *	1.49–2.24	1.35 *	1.08–1.70
Awareness of sodium labeling in restaurant or highway rest area									
Yes	263 (23.6%)	191 (25.6%)	355 (18.2%)	809 (21.2%)	<0.0001	1.56 *	1.24–1.96	0.93	0.74–1.19
Low-sodium food/goods ever used or purchased									
Low-sodium ham	444 (38.9%)	302 (41.0%)	618 (33.1%)	1364 (36.7%)	<0.0001	1.60 *	1.32–1.95	0.92	0.75–1.13
Low-sodium cereal	192 (17.2%)	90 (12.2%)	227 (12.2%)	509 (13.7%)	0.0002	1.22	0.91–1.62	1.41 *	1.06–1.89
Low-sodium ramen	261 (23.4%)	136 (18.5%)	334 (17.9%)	731 (19.7%)	0.0008	1.07	0.84–1.37	1.33 *	1.03–1.72
Sodium reduced salted fish	135 (12.1%)	114 (15.5%)	139 (7.5%)	388 (10.4%)	<0.0001	1.98 *	1.47–2.66	0.70	0.52–0.95
Sodium reduced kimchi	113 (10.1%)	77 (10.5%)	120 (6.4%)	310 (8.3%)	0.0001	1.38	0.98–1.94	0.96	0.68–1.35
Low-sodium cheese	371 (33.3%)	195 (26.5%)	428 (22.9%)	994 (26.7%)	<0.0001	1.30 *	1.05–1.62	1.33 *	1.06–1.66
Low-sodium soy sauce	307 (27.6%)	186 (25.3%)	273 (14.6%)	766 (10.6%)	<0.0001	1.77 *	1.39–2.23	1.11	0.88–1.40
Low-sodium salt	446 (40.0%)	279 (37.9%)	494 (25.5%)	1219 (32.8%)	<0.0001	2.07 *	1.69–2.54	1.01	0.82–1.24

M = Maintenance stage, A = Action stage, P = Preaction stage including the preparation, contemplation, and precontemplation stages. ^(1)^ *p* value from a chi-square test according to the stage of change group; ^(2)^ OR = Odds ratio; CI = Confidence interval. * Independently significant in multiple logistic regression models including age, sex, BMI, education level, income level (*p* < 0.05), reference is the response rate of ‘yes’ in each item; the pre-action stage is the reference group is in A versus P, and the action stage is the reference group is in M versus A; ^(3)^ *N* (%): the response rate of ‘yes’ to each item.

**Table 3:** Cognition of positive outcome expectancies and barriers to practicing according to the stages of change.

	M	A	P	Total	*p*-Value ^(1)^	A vs. P		M vs. A	
						OR ^(2)^	95% CI	OR	95% CI
Positive outcome expectancies									
Decrease of blood pressure	921 (80.0%) ^(3)^	591 (78.0%)	1400 (70.6%)	2912 (74.8%)	<0.0001	1.35 *	1.09–1.69	1.11	0.86–1.41
Prevention to stroke and heart diseases	934 (81.2%)	580 (76.5%)	1439 (72.6%)	2953 (75.8%)	<0.0001	1.14	0.91–1.42	1.25	0.98–1.60
Reduction of swelling in body	324 (28.2%)	206 (27.2%)	656 (33.1%)	1186 (30.5%)	0.0014	0.79 *	0.64–0.97	1.00	0.80–1.25
Prevention to cancer	413 (35.9%)	234 (30.9%)	637 (32.1%)	1284 (33.0%)	0.0358	0.881	0.72–1.08	1.42 *	1.14–1.76
Barriers to practice									
Bad taste	653 (62.2%)	542 (74.5%)	1639 (83.7%)	2834 (75.7%)	<0.0001	0.56 *	0.44–0.70	0.56 *	0.44–0.70
Time-consuming and inconvenient process of cooking and preparing	351 (35.1%)	334 (46.6%)	1088 (56.1%)	1773 (48.1%)	<0.0001	0.70 *	0.58–0.85	0.59 *	0.48–0.73
Limitation in choosing the food, menu, and restaurant	855 (82.6%)	595 (82.9%)	1762 (90.5%)	3212 (86.8%)	<0.0001	0.61 *	0.46–0.80	0.91	0.69–1.21
Limited information, knowledge and skills to practice	634 (62.9%)	542 (76.7%)	1660 (86.3%)	2836 (77.9%)	<0.0001	0.55 *	0.43–0.69	0.51 *	0.40–0.64
Limitation to social relationships when dining with family or friends	824 (78.7%)	591 (82.3%)	1756 (90.6%)	3171 (85.6%)	<0.0001	0.57 *	0.43–0.76	0.70 *	0.53–0.91
Preference to broth dishes (soup, stew)	567 (53.9%)	516 (70.8%)	1466 (75.0%)	2549 (68.2%)	<0.0001	0.92	0.74–1.14	0.46 *	0.37–0.57
Preference to kimchi, salted fish, fermented sauces	498 (47.5%)	484 (66.2%)	1341 (68.6%)	2323 (62.2%)	<0.0001	0.91	0.74–1.11	0.45 *	0.37–0.56

M = Maintenance stage, A = Action stage, P = Preaction stage including the preparation, contemplation, and precontemplation stages. ^(1)^ *p* value from a chi-square test according to the stage of change group; ^(2)^ OR = Odds ratio; * Independently significant in multiple logistic regression models including age, sex, BMI, education level, and income level (*p* < 0.05), the reference is the response rate of ‘yes’ in each item. The pre-action stage is the reference group is in A versus P, and the action stage is the reference group is in M versus A; ^(3)^ *N* (%): the response rate of ‘yes’ to each item.

**Table 4:** Perceptions and self-efficacy of reducing sodium intake according to the stages of change.

	M	A	P	Total	*p*-Value ^(1)^	A vs. P		M vs. A	
						OR ^(2)^	95% CI	OR	95% CI
I feel unfulfilled or unsatisfied when eating foods with less salt. ^+^	2.74 ± 0.85 ^a,^^(3)^	2.42 ± 0.76 ^b^	2.26 ± 0.66 ^c^	2.43 ± 0.77	<0.0001	1.86 *	1.53–2.27	1.86 *	1.52–2.29
I usually recognize the sodium contents in food or dishes.	3.48 ± 0.86 ^a^	3.08 ± 0.86 ^b^	2.72 ± 0.88 ^c^	3.02 ± 0.93	<0.0001	2.21 *	1.79–2.71	2.29 *	1.77–2.96
Practicing a low-sodium diet will improve my health status.	4.12 ± 0.69 ^a^	3.87 ± 0.78 ^b^	3.86 ± 0.70 ^b^	3.94 ± 0.72	<0.0001	0.69	0.45–1.07	2.24 *	1.32–3.81
I will buy fresh food rather than processed or instant food.	4.15 ± 0.83 ^a^	3.93 ± 0.86 ^b^	3.90 ± 0.75 ^b^	3.98 ± 0.81	<0.0001	0.70	0.45–1.07	1.13	0.72–1.76
I will ask to reduce the salt when eating-out.	3.46 ± 0.94 ^a^	3.17 ± 0.93 ^b^	2.93 ± 0.93 ^c^	3.13 ± 0.96	<0.0001	1.76 *	1.41–2.19	1.50 *	1.16–1.95
I will choose dishes with natural flavor and taste rather than hot, salty, spicy ones.	3.91 ± 0.82 ^a^	3.66 ± 0.84 ^b^	3.56 ± 0.82 ^c^	3.69 ± 0.84	<0.0001	1.26	0.91–1.75	1.24	0.85–1.81
I will have concern for low-sodium recipes.	4.08 ± 0.67 ^a^	3.85 ± 0.75 ^b^	3.66 ± 0.77 ^c^	3.82 ± 0.76	<0.0001	1.51	0.97–2.34	1.74 *	1.00–3.05
I think that influence of consumers’ sodium reduction can induce the change of social surroundings.	4.04 ± 0.72 ^a^	3.85 ± 0.78 ^b^	3.74 ± 0.78 ^c^	3.85 ± 0.77	<0.0001	1.49	0.96–2.32	1.26	0.75–2.12

M = Maintenance stage, A = Action stage, P = Preaction stage including the preparation, contemplation, and precontemplation stages. ^(1)^ *p* value determined by a Duncan’s multiple range test after one-way ANOVA. Different superscripts are significantly different between the groups at α = 0.05. ^(2)^ OR = Odds ratio: The reference is <3 point score respondent; * independently significant in multiple logistic regression models including age, sex, BMI, education level, and income level (*p* < 0.05). The pre-action stage is the reference group is in A versus P, and the action stage is the reference group is in M versus A. ^(3)^ Mean ± SE: Mean values adjusted by age, sex, BMI, education level, and income level from ANCOVA analysis. Score 1 = strongly disagree, 2 = disagree, 3 = neither agree nor disagree, 4 = agree, 5 = strongly agree; a higher score means better perceptions and self-efficacy. (^+^ 5 = strongly disagree, 4 = disagree, 3 = neither agree nor disagree, 2 = agree, 1 = strongly agree).

**Table 5:** The percentage of correct answers and the nutritional knowledge scores related to sodium intake according to the stages of change.

	M	A	P	Total	*p*-Value ^(1)^	A vs. P		M vs. A	
						OR ^(2)^	95% CI	OR	95% CI
Excess intake of sodium can increase the risk of osteoporosis.	892 (79.4%) ^(3)^	538 (72.0%)	1380 (70.6%)	2810 (73.5%)	<0.0001	1.11	0.90–1.37	1.38 *	1.09–1.75
The amount of sodium and the amount of salt are the same in the same food.	336 (30.0%)	247 (33.3%)	662 (33.9%)	1245 (32.7%)	0.0754	1.25 *	1.02–1.53	0.83	0.67–1.03
Two tablespoons of salt is the recommended goal intake of salt in a day.	646 (57.5%)	404 (54.7%)	870 (44.8%)	1920 (50.5%)	<0.0001	1.50 *	1.24–1.81	1.09	0.89–1.34
Sodium is necessary to keep the balance and equilibrium of body fluids.	813 (73.6%)	496 (67.6%)	1406 (72.4%)	2715 (71.8%)	0.0128	0.95	0.77–1.17	1.30 *	1.04–1.63
Sufficient intake of vegetables and fruits helps sodium excretion.	966 (86.6%)	593 (80.0%)	1591 (81.6%)	3150 (82.7%)	0.0002	0.95	0.74–1.20	1.58 *	1.20–2.09
Cooked fish with sauce contains much more salt than grilled fish in itself.	878 (79.0%)	517 (69.7%)	1412 (72.9%)	2807 (74.0%)	<0.0001	1.04	0.84–1.28	1.44 *	1.14–1.82
One tablespoon of salt contains the same amount of sodium as one tablespoon of soybean paste (miso).	588 (52.6%)	352 (47.4%)	906 (46.5%)	1846 (48.5%)	0.0048	1.10	0.91–1.33	1.17	0.96–1.44
The amount of sodium in the noodles themselves is more than that in the broth of ramen.	826 (73.3%)	495 (66.3%)	1346 (67.0%)	2667 (69.7%)	0.0031	1.06	0.87–1.30	1.33 *	1.06–1.66
Average score.	6.30 ± 0.07 ^(4)^	6.03 ± 0.09	5.79 ± 0.05	5.98 ± 0.05	<0.0001	1.01 ^(5)^	0.85–1.20	1.46 *	1.20–1.78

M = Maintenance stage, A = Action stage, P = Preaction stage including the preparation, contemplation, and precontemplation stages. ^(1)^ *p*-value from a chi-square test according to the stage of change group; ^(2)^ OR = Odds ratio: the reference is the correct answer in each item. * Independently significant in multiple logistic regression models including age, sex, BMI, education level, and income level (*p* < 0.05). The pre-action stage is the reference group is in A versus P, and the action stage is the reference group is in M versus A; ^(3)^ *N* (%): the rate of correct answer to each item. ^(4)^ Mean ± SE: Mean values adjusted by age, sex, BMI, education level, and income level from ANCOVA analysis; ^(5)^ Reference is respondent under a score of 6 (50th percentile of the average score).

**Table 6:** Dietary behaviors related to sodium intake according to the stages of change.

	M	A	P	Total	*p*-Value ^(1)^	A vs. P		M vs. A	
						OR ^(2)^	95% CI	OR	95% CI
I often eat dried fish, salted and fermented fish, and salted mackerel	991 (87.4%) ^(3)^	645 (86.5%)	1613 (82.1%)	3249 (84.5%)	0.0001	1.34 *	1.07–1.82	1.07	0.80–1.45
I often eat processed or instant food such as ramen, ham, and canned food.	1009 (89.9%)	654 (88.6%)	1534 (78.3%)	3197 (83.7%)	<0.0001	1.93 *	1.44–2.58	0.96	0.69–1.35
I add salt or sauces more when eating bland tasting dishes.	857 (75.7%)	471 (63.1%)	1073 (54.7%)	2401 (62.5%)	<0.0001	1.64 *	1.35–1.99	1.72 *	1.38–2.15
I eat all of the soup, stew, broth, or noodle liquid.	833 (73.7%)	518 (70.0%)	1236 (63.5%)	2587 (67.8%)	<0.0001	1.34 *	1.09–1.64	1.17	0.94–1.46
I frequently eat soy paste soup or other broth soups and stew (Jjigae, jeongol).	809 (71.8%)	502 (68.0%)	1169 (59.9%)	2480 (65.0%)	<0.0001	1.57 *	1.32–1.87	1.38 *	1.13–1.69
I eat out (including delivery foods) or have a dining meeting more than two or three times in a week.	903 (81.0%)	599 (81.4%)	1428 (73.1%)	2930 (77.0%)	<0.0001	1.38 *	1.09–1.74	0.97	0.75–1.26
I usually eat fried or pan-fried dishes and sliced raw fish with plenty of dipping sauces.	964 (85.8%)	602 (81.5%)	1430 (73.3%)	2996 (78.6%)	<0.0001	1.45 *	1.15–1.83	1.38 *	1.05–1.80
I prefer braised fish with soy sauce than fresh grilled fish.	864 (77.1%)	540 (73.3%)	1360 (69.6%)	2764 (72.5%)	<0.0001	1.23 *	1.00–1.53	1.26 *	1.00–1.56
I often eat plenty of fruits and vegetables. ^+^	1055 (94.5%)	647 (89.0%)	1696 (87.3%)	3398 (89.8%)	<0.0001	1.32	0.97–1.80	1.99 *	1.34–2.95
I usually check the sodium content in nutrition labeling when eating-out or purchasing food. ^+^	663 (58.9%)	361 (48.7%)	673 (34.5%)	1697 (44.5%)	<0.0001	1.87 *	1.54–2.26	1.45 *	1.19–1.78
I often eat potato chips or crackers as a snack.	1038 (92.1%)	671 (90.2%)	1646 (84.0%)	3355 (87.6%)	<0.0001	1.60 *	1.19–2.16	1.22	0.86–1.73
Average score.	893 (86.0%)	502 (18.6%)	1188 (63.8%)	2583 (72.5%)	<0.0001	1.60 *	1.28–1.99	1.96 *	1.50–2.56

M = Maintenance stage, A = Action stage, P = Preaction stage including the preparation, contemplation, and precontemplation stages. ^(1)^ *p* value from a chi-square test according to the stage of change group; ^(2)^ OR = Odds ratio: * Independently significant in multiple logistic regression models including age, sex, BMI, education level, and income level (*p* < 0.05). The pre-action stage is the reference group is in A versus P, and the action stage is the reference group is in M versus A. The reference is respondent over three points in each item; ^(3)^ *N* (%): the response rate is over three points (score range = 1~5; 5 = strongly disagree, 4 = disagree, 3 = neither agree nor disagree, 2 = agree, 1 = strongly agree); a higher score means better dietary pattern related sodium intake, (^+^ 1 = strongly disagree, 2 = disagree, 3 = neither agree nor disagree, 4 = agree, 5 = strongly agree).

## Supplementary Material

The following are available online at www.mdpi.com/2072-6643/9/8/808/s1.

## References

[1] He F.J., Li J., MacGregor G.A. (2013). Effects of longer term modest salt reduction on blood pressure. Cochrane systematic review and meta-analysis of randomized trials. BMJ.

[2] Mozaffarian D., Fahimi P.H., Singh G.M., Micha R., Khatibzadeh S., Engell R.E., Lim S., Danaei G., Ezzati M., Powles J. (2014). Global sodium consumption and death from cardiovascular causes. N. Engl. J. Med..

[3] Antonios T.F., MacGregor G.A. (1995). Deleterious effects of salt intake other than effects on blood pressure. Clin. Exp. Pharmacol. Physiol..

[4] Webster J.L., Dunford E.K., Hawkes C., Neal B. (2011). Salt reduction initiatives around the world. J. Hypertens..

[5] Asaria P., Chrisholm D., Mathers C., Ezzati M., Beaglehole R. (2007). Chronic disease prevention: Health effects and financial costs of strategies to reduce salt intake and control tobacco use. Lancet.

[6] Ministry of Health and Welfare, Korea Centers for Disease Control and Prevention (2015). Korea Health Statistics 2014: Korea National Health and Nutrition Examination Survey (KNHANES V-2).

[7] Shankar B., Brambila-Macias J., Traill B., Mazzocchi M., Capacci S. (2013). An evaluation of the UK Food Standards Agency’s salt campaign. Health Econ..

[8] Nissinen A., Kastarinen M., Tuomilehto J. (2004). Community control of hypertension-experiences from Finland. J. Hum. Hypertens..

[9] Paik H.Y. (1987). Nutritional review of salt. J. Korean Soc. Food Sci. Nutr..

[10] Prohaska J.O., Velicer W.F. (1997). The transtheoretical model of health behavior change. Am. J. Health Promot..

[11] Heather N., Rollnick S., Bell A. (1993). Predictive validity of the readiness to change questionnaire. Addiction.

[12] Prochaska J.O., Redding C.A., Harlow L.L., Rossi J.S., Velicer W.F. (1994). The transtheoretical model of change and HIV prevention: A review. Health Educ. Q..

[13] Newson R.S., Elmadfa I., Biro G., Cheng Y., Prakash V., Rust P., Barna M., Lion R., Meijer G.W., Neufingeri N. (2013). Barriers for progress in salt reduction in the general population. An international study. Appetite.

[14] Baranowski T., Perry C.L., Parcel G., Glanz K., Rimer B.K., Lewis F.M. (2002). How individuals, environments, and health behaviors interact: Social cognitive theory. Health Behavior and Health Education: Theory, Research and Practice.

[15] Hearn M., Baranowski T., Baranowski J., Doyle C., Smith M., Lin L.S., Resnicow K. (1998). Environmental influences on dietary behavior among children: Availability and accessibility of fruits and vegetables enable consumption. J. Health Educ..

[16] Jung E.J., Son S.M., Kwon J.S. (2012). The effect of sodium reduction education program of a public health center on the blood pressure, blood biochemical profile and sodium intake of hypertensive adults. Korean J. Community Nutr..

[17] Son S.M., Lee K.H., Kim K.W., Lee Y.K. (2007). Nutrition Education and Counseling Practice.

[18] Yim K.S. (2008). The effects of a nutrition education program for hypertensive female elderly at the public health center. Korean J. Community Nutr..

[19] Grimes C.A., Riddell L.J., Nowson C.A. (2009). Consumer knowledge and attitudes to salt intake and labelled salt information. Appetite.

[20] Kim M.K., Lopetcharat K., Gerard P.D., Drake M.A. (2012). Consumer awareness of salt and sodium reduction and sodium labeling. J. Food Sci..

[21] World Health Organization (2010). Creating an Enabling Environment for Population-Based Salt Reduction Strategies: Report of a Joint Technical Meeting Held by WHO and the Food Standards Agency, United Kingdom, July 2010.

[22] Kim M.K., Lee K.G. (2014). Consumer awareness and interest toward sodium reduction trends in Korea. J. Food Sci..

[23] Sarmugam R., Worsley A., Flood V. (2014). Development and validation of a salt knowledge questionnaire. Public Health Nutr..

[24] Zhang J., Xu A.Q., Ma J.X., Shi X.M., Guo X.L., Engelgau M., Yan L.X., Li Y., Li Y.C., Wang H.C. (2013). Dietary sodium intake: Knowledge, attitudes and practices in Shandong province, China, 2011. PLoS ONE.

[25] Yon M.Y., Lee Y.N., Kim D.H., Lee J.Y., Koh E.M., Nam E.J., Shin H.H., Kang B.W., Kim K.W., Heo S. (2011). Major sources of sodium intake of the Korean population at prepared dish level -Based on the KNHANES 2008 & 2009. Korean J. Community Nutr..

[26] Sultan S., Anjum F.M., Butt M.S., Huma N., Suleria H.A. (2014). Concept of double salt fortification; a tool to curtail micronutrient deficiencies and improve health status. J. Sci. Food Agric..

[27] Marakis G., Tsigarida E., Mila S., Panagiotakos D.B. (2014). Knowledge, attitudes and behaviour of Greek adults towards salt consumption: A Hellenic food authority project. Public Health Nutr..

[28] Webster J.L., Li N., Dunford E.K., Nowson C.A., Neal B.C. (2010). Consumer awareness and self-reported behaviours related to salt consumption in Australia. Asia Pac. J. Clin. Nutr..

[29] Land M.A., Webster J., Christoforou A., Johnson C., Trevena H., Hodgins F., Chalmers J., Woodward M., Barzi F., Smith W. (2014). The association of knowledge, attitudes and behaviors related to salt with 24-hour urinary sodium excretion. Int. J. Behav. Nutr. Phys. Act..

[30] Okuda N., Stamler J., Brown I.J., Ueshima H., Miura K., Okayama A., Saitoh S., Nakagawa H., Sakata K., Yoshita K. (2014). Individual efforts to reduce salt intake in China, Japan, UK, USA: What did people achieve? The INTERMAP Population Study. J. Hypertens..

[31] Ortega R.M., López-Sobaler A.M., Ballesteros J.M., Pérez-Farinós N., Rodriguez-Rodriguez E., Aparicio A., Perea J.M., Andrés P. (2011). Estimation of salt intake by 24 h urinary sodium excretion in a representative sample of Spanish adults. Br. J. Nutr..

[32] Sarmugam R., Worsley A. (2014). Current levels of salt knowledge: A review of the literature. Nutrients.

[33] Diclemente C., Prohaska J., Miller W.R., Heather N. (1998). Toward a comprehensive, transtheoretical model of change. Treating Addictive Behaviours.

[34] Sheeshka J.D., Woolcott D.M., MacKinnon N.J. (1993). Social cognitive theory as a framework to explain intentions to practice health eating behaviors. J. Appl. Soc. Psychol..

