# Supplementary material for: Stages of Behavioral Change for Reducing Sodium Intake in Korean Consumers: Comparison of Characteristics Based on Social Cognitive Theory
Source: Nutrients. 2017 Jul 27;9(8):808. doi: 10.3390/nu9080808 (PMC5579602; doi:10.3390/nu9080808)
Supplement: Supplementary file 1 [file nutrients-09-00808-s001.zip › nutrients-207532-supplementary.pdf]

Thank you for participating this survey. It aims to investigate the status of Korean dietary life. Your response will be only used for survey purposes, and all your answers will be held in the strictest confidentially.

Please put a tick in the box ☑ next to the answer of your choice or write in the space provided.

1. Are you currently practicing a low sodium diet?

   ☐ Yes, more than six months

   ☐ Yes, but less than six months

   ☐ No (Please, answer the next question)

   1-1. Do you intend to make changes to reduce your sodium intake in the near future or in the following month?

   ☐ Yes

   ☐ No, but in the next six months,

   ☐ No, I haven't thought about it.

2. Do you recognize any effort (nutrition education program, campaign, or events) to encourage the reduction sodium intakes in schools, government, food service industries, TV, radio, advertisements, and newspapers?

   ☐ Yes ☐ No

3. Have you seen nutritional labeling of sodium on processed foods?

   ☐ Yes ☐ No

4. Do you know about the nutritional labeling of sodium in restaurants or highway rest areas?

   ☐ Yes ☐ No

5. Please check the list of low-sodium foods ever used or purchased (multiple answers possible).

   ☐ Low-sodium ham
   ☐ Low-sodium snack
   ☐ Low-sodium cereal
   ☐ Low-sodium ramen
   ☐ Sodium-reduced salted fish
   ☐ Sodium reduced kimchi
   ☐ Low-sodium cheese
   ☐ Low-sodium soy sauce
   ☐ Low-sodium salt

6. What advantages do you expect from reducing your sodium intake? (3 choices possible)

   ☐ Decrease of blood pressure
   ☐ Prevention of stroke and heart diseases
   ☐ Weight loss
   ☐ Reduction of swelling in body
   ☐ Skin enhancement
   ☐ Prevention of osteoporosis
   ☐ Prevention of cancer

7. Please rate the following statements regarding difficulties to practicing a low-sodium diet.

	Strongly disagree	Disagree	Neither agree nor disagree	agree	Strongly agree
I have difficulties in practicing a low sodium-diet because of					
Bad taste	☐	☐	☐	☐	☐
Time-consuming and inconvenient process of cooking and preparing	☐	☐	☐	☐	☐
Limitation in choosing the food, menu and restaurant	☐	☐	☐	☐	☐
Limited information, knowledge, and skills to practice	☐	☐	☐	☐	☐
Limitation to social relationships when dining with family or friends	☐	☐	☐	☐	☐
Preference for broth dishes (soup, stew)	☐	☐	☐	☐	☐
Preference for kimchi, salted fish, fermented sauces	☐	☐	☐	☐	☐

8. Please rate the following statements regarding your dietary habit.

	Strongly disagree	Disagree	Neither agree nor disagree	agree	Strongly agree
I often eat dried fish, salted and fermented fish, and salted mackerel	☐	☐	☐	☐	☐
I often eat processed or instant food such as ramen, ham, and canned food.	☐	☐	☐	☐	☐
I add salt or sauces more when eating bland tasting dishes.	☐	☐	☐	☐	☐
I eat all of the soup, stew, broth or noodle liquid.	☐	☐	☐	☐	☐
I frequently eat soy paste soup or other broth soups and stews (Jjigae, eongol).	☐	☐	☐	☐	☐
I eat out (including delivery foods) or have a dining meeting more than two or three times in a week.	☐	☐	☐	☐	☐
I eat kimchi frequently.	☐	☐	☐	☐	☐
I usually eat fried or pan-fried dishes and sliced raw fish with plenty of dipping sauces.	☐	☐	☐	☐	☐
I prefer braised fish with soy sauce to fresh grilled fish.	☐	☐	☐	☐	☐
I often eat plenty of fruits and vegetables.	☐	☐	☐	☐	☐
I usually check the sodium content in nutrition labeling when eating-out or purchasing food.	☐	☐	☐	☐	☐
I usually buy seasoned meat or seafood and dishes for sale.	☐	☐	☐	☐	☐
I often eat potato chips or crackers as a snack.	☐	☐	☐	☐	☐

9. Please indicate your level of agreement with the following statement.

	Strongly disagree	Disagree	Neither agree nor disagree	agree	Strongly agree
I feel unfulfilled or unsatisfied when eating foods with less salt.	☐	☐	☐	☐	☐
I usually recognize the sodium contents in food or dishes.	☐	☐	☐	☐	☐
Practicing a low-sodium diet will improve my health status.	☐	☐	☐	☐	☐
I will buy fresh food rather than processed or instant food.	☐	☐	☐	☐	☐
I will ask to reduce the salt when eating-out.	☐	☐	☐	☐	☐
I will choose dishes with natural flavor and taste rather than hot, salty, spicy ones.	☐	☐	☐	☐	☐
I will have concern for low-sodium recipes.	☐	☐	☐	☐	☐
I think that the influence of consumers' sodium reduction can induce the change of social surroundings.	☐	☐	☐	☐	☐

10. Please select answer of the following questions.

	Yes	No	Don't know
An excess intake of sodium can increase the risk of osteoporosis.	☐	☐	☐
The amount of sodium and the amount of salt are the same in the same food.	☐	☐	☐
Two tablespoons of salt are the recommended goal intake of salt in a day.	☐	☐	☐
Sodium is necessary to keep the balance and equilibrium of body fluids.	☐	☐	☐
Sodium exists in various food additives such as baking powder and preservatives.	☐	☐	☐
A sufficient intake of vegetables and fruits helps sodium excretion.	☐	☐	☐
For nutrition labeling, salt content is indicated in the labeling.	☐	☐	☐
Cooked fish with sauce contains much more salt than grilled fish in itself.	☐	☐	☐
One tablespoon of salt contains the same amount of sodium as one tablespoon of soybean paste (miso).	☐	☐	☐
The amount of sodium in the noodles themselves is more than that in the broth of ramen.	☐	☐	☐

11. Please fill out the following for your personal information.

Gender	☐ Male ☐ Female
Age	( ) years old
Height	( ) cm
Weight	( ) kg
Residence	☐ Major city<br>☐ Small and medium-sized city<br>☐ Rural community
Education qualification	☐ Middle school or less<br>☐ High school<br>☐ College or above
Average monthly income	☐ Under $1,000<br>☐ $1,000-$2,000<br>☐ $2,00-$3,00<br>☐ $3,000-$4,000<br>☐ $4,000-$5,000<br>☐ Above $5,000

*Thank you for completing the survey.
